# Novel Natural Mutations in the Hepatitis B Virus Reverse Transcriptase Domain Associated with Hepatocellular Carcinoma

**DOI:** 10.1371/journal.pone.0094864

**Published:** 2014-05-01

**Authors:** Yan Wu, Yu Gan, Fumin Gao, Zhimei Zhao, Yan Jin, Yu Zhu, Zhihan Sun, Hao Wu, Taoyang Chen, Jinbing Wang, Yan Sun, Chunsun Fan, Yongbing Xiang, Gengsun Qian, John D. Groopman, Jianren Gu, Hong Tu

**Affiliations:** 1 State Key Laboratory of Oncogenes and Related Genes, Shanghai Cancer Institute, Renji Hospital, Shanghai Jiao Tong University School of Medicine, Shanghai, China; 2 Department of Epidemiology, School of Public Health, Fudan University, Shanghai, China; 3 School of Basic Medical Sciences, Fudan University, Shanghai, China; 4 Department of Etiology, Qidong Liver Cancer Institute/Qidong People’s Hospital, Qidong, Jiangsu, China; 5 Bloomberg School of Public Health, Johns Hopkins University, Baltimore, Maryland, United States of America; University of Pisa, Italy

## Abstract

**Background/Aim:**

Hepatitis B Virus (HBV) mutations play a role in the development of hepatocellular carcinoma (HCC). However, the association between HBV polymerase gene mutations and HCC has not been reported. In this study, we conducted a multi-stage study to identify HCC-related mutations in the reverse transcriptase (RT) domain of the HBV polymerase gene.

**Methods:**

A total of 231 HCCs and 237 non-HCC controls from Qidong, China, were included in this study. The entire sequence of HBV RT was first compared between 29 HCC and 35 non-HCC cases, and candidate mutations were then evaluated in two independent validation sets.

**Results:**

There were 15 candidate mutations identified from the discovery set, with A799G and T1055A being consistently associated with HCC across all studies. A pooled analysis of samples revealed that A799G, A987G, and T1055A were independent risk factors for HCC, with adjusted odds ratios of 5.53 [95% confidence interval (CI), 1.69–18.10], 4.20 (95%CI, 1.15–15.35), and 3.78 (95%CI, 1.45–9.86), respectively. A longitudinal study showed that these mutations were detectable 4–5 years prior to HCC diagnosis.

**Conclusions:**

Our study provides evidence the first that HBV RT contains naturally occurring mutations that can be used as predictive markers for HCC.

## Introduction

Hepatitis B virus (HBV) infection is one of the most common public health problems in the world. An estimated 2 billion people are infected with HBV and 360 million people worldwide have chronic HBV (CHB) infection [1], which is one of the major etiologic factors for hepatocellular carcinoma (HCC). The HBV genome contains four overlapping open reading frames (ORFs), encoding the viral polymerase, envelope protein, X protein, and nucleocapsid [2]. Although HBV is a DNA virus, its replication occurs through RNA-intermediated reverse transcription, and mutations occur at a relatively high frequency because the HBV reverse transcriptase (RT) does not have a proofreading function. Indeed, it has been estimated that approximately 10^10–11^ point mutations can be produced per day in individuals with active replication [3]. Therefore, various mutations accumulate across the HBV genome during the long-term history of viral infection. In past decades, many studies have focused on the association of HBV mutation and HCC risk. A recent meta-analysis including a total of 11,582 HBV-infected participants revealed that deletions in the pre-S gene and mutations of C1653T, T1753V and A1762T/G1764A in the basal core promoter (BCP)/enhancer II (EnhII) region had statistically significant summary odds ratios (OR) for HCC [4]. In addition, A2159G and A2189C in the core gene [5] and G1896A [6] and G1899A [7] in the pre-C gene have also been reported to increase the risk of HCC. Many recent studies have found that combined mutations involving different HBV genes regions have a stronger association with HCC than the single mutations [7]–[9]. Nonetheless, the relationship between HBV polymerase (P) gene mutations and HCC risk has not yet been elucidated.

The HBV P gene, the longest ORF within the HBV genome and encoding an 843-amino acid (aa) polypeptide, has 4 domains: a priming region (aa 1–177), a spacer region of unknown function (aa 178–346), a catalytic region (RT domain) that functions as an RNA-dependent RNA polymerase/DNA polymerase (aa 347–690), and a carboxy terminal region (aa 691–843) that has ribonuclease H activity. Based on the structure deduced from the human immunodeficiency virus-1 RT [10], the catalytic region can be subdivided into 7 domains, namely, A–G. Domain A (rt 75–91) forms part of the dNTP binding pocket and is involved in the coordination of the incoming triphosphate moiety of dNTP, whereas domain B (rt 163–189) forms a helix with a loop region and is involved in positioning the primer-template strand to the catalytic region. Domain C (rt 200–210) contains a conserved motif of tyrosine-methionine-aspartate-aspartate (YMDD), which represents the active site of the enzyme. Residues within domain D (rt 230–241) may contribute to the dNTP binding site, and domain E (rt 247–257) forms part of the template-primer binding site. Domains F (rt 37–47) and G (rt 26–36) are upstream of domain A and may be involved in interactions with the incoming dNTP and also with the template nucleotide (nt). HBV RT is essential for the production of new HBV-DNA containing nucleocapsids, and hence virions, and is the major molecular target for anti-HBV development [11].

Nucleoside analogs (NAs), i.e., lamivudine, telbivudine, adefovir dipivoxil, tenofovir disoproxil fumarate, and entecavir, have been used to treat CHB infection in many parts of the world. Although these NAs successfully inhibit viral replication *in vivo*, they also progressively induce resistance mutations in HBV RT over the course of treatment. Two types of mutations are associated with treatment failure to NA: primary mutations, which are directly responsible for drug resistance, and compensatory mutations enhancing replication ability [12], [13]. For example, resistance to lamivudine and telbivudine is conferred by mutation rtM204V or rtM204I in the YMDD motif, and this is often associated with compensatory mutations rtL180M and/or rtV173L restoring a higher replication capacity [13]. Although long-term treatment with NA may reduce the development of HCC [14], [15], data regarding the relationship between drug-resistance mutation and HCC development are limited.

Qidong, China is the area that is most affected by HCC worldwide, with an age-standardized incidence rate of 77.7 per 100,000 for males and 24.3 per 100,000 for females [16]. HCC is the leading cause of cancer mortality and accounts for almost half of the cancer occurrence in Qidong, and HBV infection poses a high level of risk for HCC in Qidong [OR: 11.7; 95% confidence interval (CI): 9.1–15.2] [17]. Over the past few years, we defined that a high prevalence of the HBV C1 genotype [18], pre-S deletion and pre-S2 start codon mutation [19], C1653T, T1753A/G, C1766T, and T1768A mutations in the BCP/EnhII region [20], [21], and A2159G, A2189C, and G2203W in the core gene [5] are associated with HCC in Qidong. Notably, using serial samples collected from a prospective cohort, we observed for the first time that mutations in the BCP/Enh II region occurred in a temporal order during the course of HCC development [20]. In the present study, we extended our research to the HBV RT domain. After comparing the RT sequences from 231 HCC cases and 237 non-HCC controls, we identified three novel mutations significantly associated with HCC.

## Methods

### Participants and Study Design

**Figure 1:**
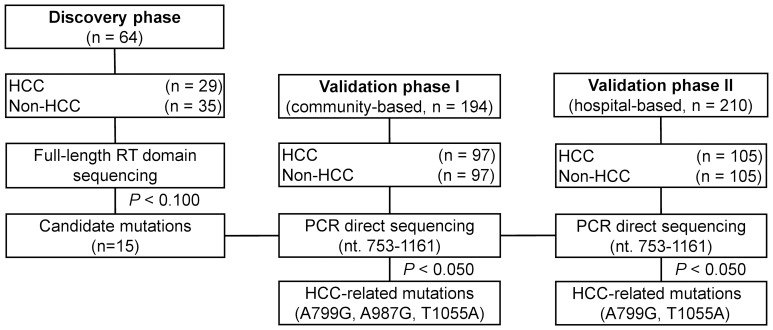
Study design. Abbreviations: HCC, hepatocellular carcinoma; PCR, polymerase chain reaction; RT, reverse transcriptase.

The HCC cases and HBV-infected non-HCC controls for the discovery set and validation set I were obtained from a prospective cohort starting in 1992, including 807 hepatitis B surface antigen (HBsAg)-positive and 761 HBsAg-negative individuals from 7 towns of Qidong [22]. Blood samples were collected annually for each of the cohort members. None of the subjects in this cohort received treatment with NAs or interferon. The patients in validation set II were obtained from Qidong People’s Hospital between October 2007 and October 2012. We carefully checked the participants’ personal information, including ID number, name, gender, address, and date of birth, and did not find any duplicate entry in our study. The participants of these two validation sets have been frequency-matched for both age and sex between HCC cases and non-HCC controls. Since all participants in the discovery set were infected with genotype C virus determined by phylogenetic tree analysis, we only included genotype C HBV-infected patients in the two validation sets. The diagnosis of HCC was based on one of the following criteria: (1) positive histological findings or (2) positive imaging findings on either computerized tomography or ultrasonography along with α-fetoprotein serum levels of ≥400 ng/mL. The flow chart of the study procedure is demonstrated in Figure 1. Briefly, in the discovery set, the entire sequence of HBV RT was compared among 29 HCC and 35 non-HCC individuals. The mutations that showed a differential incidence rate between HCCs and non-HCCs with a *P* value lower than 0.100 were selected as candidates for further validation in two independent sets. The study was approved by the local ethics committee at Qidong People’s Hospital and Shanghai Cancer Institute and conducted according to the principles of the Declaration of Helsinki. Written informed consent was signed by each participant.

### Amplification and Sequencing of the HBV RT Domain

The QIAamp MinElute Virus Spin Kit (QIAGEN, Hilden, Germany) was used to extract HBV DNA from 100 µl plasma according to the manufacturer’s protocol. For the discovery set, the entire sequence of HBV RT domain (nt 130–1161) was amplified by semi-nested polymerase chain reaction (PCR) using HBV57F and 1281R as the first-round primers and HBV57F and 1193R2 as the second-round primers. For the validation set, the 3′-terminal sequence of RT (nt 753–1161) was amplified by nested PCR using FLP6 and 1281R as the first-round primers and HBV696F and 1193R2 as the second-round primers. The primer sequences are listed in Table S1 in File S1. PrimeSTAR Max polymerase (Takara Biotechnology Dalian, Dalian, China) was used in all PCRs, and the PCR products were gel-purified and subsequently sent for automated sequencing (Biosune, Shanghai, China). BioEdit software (version 5.0.9; available at http://www.mbio.ncsu.edu/BioEdit/bioedit.html) was used to align the validated sequences with respect to the prototype sequence of HBV genotype C (GenBank Accession No. EU871982.1). A total of 468 sequences in the RT region of HBV polymerase determined in this study have been deposited in GenBank. The corresponding accession numbers are from KJ460502 to KJ460969.

### Serologic Assay

HBsAg and hepatitis B e antigen (HBeAg) were determined by commercially available kits (Kehua, Shanghai, China).

### HBV Genotyping

The HBV genotypes were determined by comparing the sequences of HBV RT with different HBV genotype (A to H) sequences retrieved from GenBank as reference genes. A phylogenetic tree was constructed using MEGA 4.1 software [23].

### Quantification of Circulating HBV DNA

The level of circulating HBV DNA was quantified by real-time PCR using a commercial HBV diagnostic kit (PG Biotech, Shenzhen, China), as described previously [20]. The detection limit of the kit was 5.0×10^2^ copies/mL.

### Statistical Analysis

The chi-square test or Fisher’s exact test was used to analyze the categorical variables. For the circulating HBV DNA data (log_10_ copies/mL, presented as the mean ± SE) obtained by real-time PCR, the statistical analyses were performed using Student’s *t*-test. The crude ORs and 95% CIs were calculated using a two-by-two frequency table. Multivariate unconditional logistic regression analyses were used to estimate the adjusted ORs and 95% CIs. All of the tests were two-tailed, and a *P* value of <0.050 was considered to be statistically significant. The statistical analysis was performed using SPSS software version 12.0 (SPSS Inc., Chicago, IL, USA).

## Results

### Patient Characteristics

The characteristics of the participants in discovery set and two validation sets are shown in Table 1. The distributions of age and sex were matched between the HCC and non-HCC groups in all sets. There was no significant difference in HBeAg positivity and circulating HBV DNA level between the HCC cases and non-HCC controls in any of the three sets (all *P*>0.050). However, there were more patients with abnormal liver function (ALT >40 IU/L) in the HCC group than in the non-HCC group in all sets (both *P*<0.001).

**Table 1:** Characteristics of the study participants in the validation datasets.

	Discovery set			Validation set I			Validation set II		
Characteristic	HCC (n = 29)*	Non-HCC (n = 35)*	*P*	HCC (n = 97)*	Non-HCC (n = 97)*	*P*	HCC (n = 105)*	Non-HCC (n = 105)*	*P*
Age, mean ± SD	51.9±8.9.	52.2±9.4	Matched	52.6±12.5	52.0±9.0	Matched	49.8±9.7	48.7±9.5	Matched
Sex, male	22 (75.9)	26 (74.3)	Matched	73 (75.3)	74 (76.3)	Matched	90 (85.7)	90 (85.7)	Matched
HBeAg positive	11 (37.9)	8 (22.9)	0.189	32 (33.3)	22 (22.9)	0.108	39 (37.1)	28 (26.7)	0.103
Serum HBV DNA level (log_10_ copies/mL ≥5)	15 (51.7)	17 (48.6)	0.802	49 (50.5)	46 (47.4)	0.667	56 (53.3)	58 (55.2)	0.782
Serum ATL level (>40 IU/L)	17 (58.6)	3 (8.6)	<0.001	67(69.1)	6 (6.2)	<0.001	37 (35.2)	9 (8.6)	<0.001

Abbreviations: ALT, alanine aminotransferase; HBV, hepatitis B virus; HBeAg, hepatitis B e antigen; HCC, hepatocellular carcinoma.

*Data are presented as *n* (%), unless otherwise indicated.

### Distribution of Naturally Occurring Mutations in HBV RT

**Figure 2:**
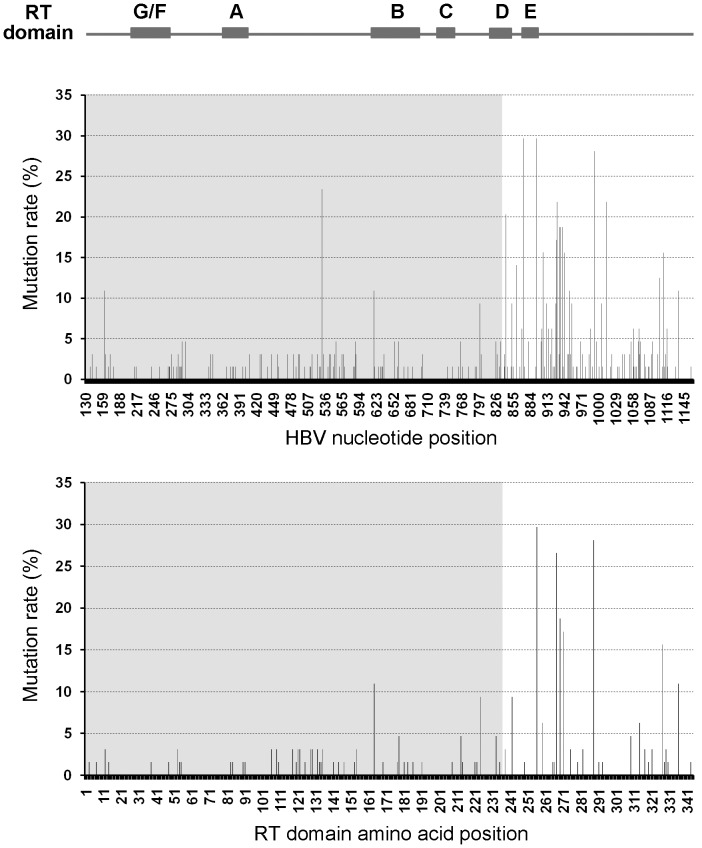
Frequency of nucleotide mutations and amino acid substitutions within HBV RT domain (*n* = 64). The values are presented as the percent nucleotide mutations (upper panel) or amino acid substitution (lower panel) compared to the wild-type genotype C hepatitis B virus (HBV) DNA sequence. The locations of the domains (A–F) are shown at the top of the figure. The pre-S/S-overlapping region in the polymerase gene is identified by grey areas. Abbreviation: HBV, hepatitis B virus; RT, reverse transcriptase.

DNA sequences of the HBV RT domain(nt 130–1161)were determined by direct PCR sequencing using the samples from 29 HCC patients and 35 HBV carriers. According to the results of the phylogenetic tree, all of these subjects were infected with the genotype C virus. Thus, a prototype sequence of HBV genotype C from a CHB patient was used as the reference to perform the alignment. A total of 101 nucleotide mutations were found in the HBV RT domain. As depicted in Figure 2, the majority of these mutation sites were located in the 3′-terminal region of RT (nt 836–1161), which does not overlap with the HBV preS2/S gene (nt 3205–835). The average mutation rate in this RT 3′-terminal region was significantly higher than that of the RT 5′-terminal region spanning from nt 130 to nt 835 (for HCC, 16.9±8.7 per 1000 nts vs. 4.4±2.6 per 1000 nts, *P*<0.001; for non-HCC, 17.7±12.6 per 1000 nts vs. 3.5±2.6 per 1000 nts, *P*<0.001). At the protein level, the total number of aa substitution sites was 80; the distribution and frequency of the aa substitutions in the RT domain are presented in Table S2 in File S1. The conservative-function G domain showed no aa substitutions, whereas domains A, B, C, D, E and F exhibited 4, 7, 1, 3, 2, and 1 substitution, respectively. After normalization with the size of each domain, domain E displayed the highest aa substitution rate (20.41 per 1000 aa), followed by the region from domain E to the end of RT (18.86 per 1000 aa) and the space between domain D and domain E (18.75 per 1000 aa). It is worth noting that we did not observe any of the common NA-related resistance mutations including rtM204V/I, rtS202C/G/I, rtL180M, rtA181T/V, rtT184A/I/L/G/C/M, rtA194T, rtI169T, rtV173L, rtL80I, rtN236T, and rtM250I/V [12], [13], [24], suggesting that these variants were either absent or present at a very low frequency in our study subjects.

### Identification of HCC-related RT Mutations

There were 15 mutations that tended to show a borderline significant difference in the mutation rate between the HCC and control groups (*P*<0.100, Table 2). Except for T906R and A942S, all of the mutations occurred with a higher frequency in the HCC patients than the non-HCC controls. These mutations resulted in six aa changes in the P gene ORF and four aa changes in the S gene ORF. Because this study aimed to investigate the effect of RT mutations on HCC, we then selected 11 mutations (A799G, T843C, T870C, T895A, T906R, C936T, A942S, A987G, T1055A, T1069A, and T1071C) that did not affect HBsAg function as candidates for further validation assays.

**Table 2:** Discovery of candidate mutations associated with hepatocellular carcinoma in the reverse transcriptase domain of hepatitis B virus.

	Amino acid change					
Nucleotide mutation	P ORF	S ORF	Location of RT domain	HCC (n = 29)*	Non-HCC (n = 35)*	*P*
C294T	NC (NC)	T48M	Space F-A	3 (10.3)	0 (0.0)	0.088
C300G	NC (NC)	P49R	Space F-A	3 (10.3)	0 (0.0)	0.088
T555C	NC (NC)	F134S	Space B-C	3 (10.3)	0 (0.0)	0.088
T766A	S559T (rtS213T)	S204R	Space C-D	3 (10.3)	0 (0.0)	0.088
A799G	I570V (rtI224V)	NC	Space C-D	6 (20.7)	0 (0.0)	0.006
T843C	NC (NC)	NA	Domain E	9 (31.0)	4 (11.4)	0.066
T870C	NC (NC)	NA	Domain E	4 (13.8)	0 (0.0)	0.037
T895A	C620S (rtC256S)	NA	Space E-end	12 (41.4)	7 (20.0)	0.098
T906R	NC (NC)	NA	Space E-end	2 (6.9)	8 (22.9)	0.097
C936T	NC (NC)	NA	Space E-end	9 (31.0)	3 (8.6)	0.028
A942S	H617Q (rtH271Q)	NA	Space E-end	2 (6.9)	8 (22.9)	0.097
A987G	NC (NC)	NA	Space E-end	6 (20.7)	1 (2.9)	0.061
T1055A	M655K (rtM309K)	NA	Space E-end	3 (10.3)	0 (0.0)	0.088
T1069A	C660S (C314S)	NA	Space E-end	4 (13.8)	0 (0.0)	0.037
T1071C	NC (NC)	NA	Space E-end	3 (10.3)	0 (0.0)	0.088

Abbreviations: NA, not available, NC, not changed; rt, reverse transcriptase; P, polymerase; S, surface antigen; ORF, open reading frame.

*Data are presented as *n* (%).

### Validation of HCC-related RT Mutations

To evaluate the association of HBV RT mutations and HCC, the 11 candidate mutations identified from the full-length RT sequence comparison study were analyzed in an independent set composed of 97 HCC patients and 97 non-HCC controls from a community-based cohort. Three mutations, A799G (15.5% in HCC vs. 5.2% in non-HCC, *P* = 0.018), A987G (11.3% in HCC vs. 2.1% in non-HCC, *P = *0.010) and T1055A (22.7% in HCC vs. 6.2% in non-HCC, *P = *0.001), showed a significantly higher frequency in the HCC group than that in the non-HCC group (Table 3). The crude ORs for A799G, A987G and T1055A were 3.37 (95% CI: 1.17–9.67), 6.08 (95%CI: 1.31–28.19) and 4.45 (95% CI: 1.72–11.54), respectively. A799G and T1055A are missense mutations resulting in aa substitutions of I224V and M309K, and A987G is a synonymous mutation.

**Table 3:** Validation of hepatocellular carcinoma-related mutations in the reverse transcriptase domain of hepatitis B virus polymerase.

	Validation set I				Validation set II			
Mutation	HCC (n = 97)*	Non-HCC (n = 97)*	*P*	Crude OR (95% CI) †	HCC (n = 105)*	Non-HCC (n = 105)*	*P*	Crude OR (95% CI) †
A799G	15 (15.5)	5 (5.2)	0.018	3.37 (1.17–9.67)	15 (14.3)	6 (5.7)	0.038	2.75 (1.02–7.39)
T843C	16 (16.5)	26 (26.8)	0.081	0.54 (0.27–1.09)	31 (29.5)	32 (30.5)	0.880	0.96 (0.53–1.73)
T870C	7 (7.2)	5 (5.2)	0.551	1.43 (0.44–4.68)	10 (9.5)	6 (5.7)	0.298	1.74 (0.61–4.97)
T895A	48 (49.5)	43 (44.3)	0.472	1.23 (0.70–2.16)	58 (55.2)	53 (50.5)	0.489	1.21 (0.70–2.08)
T906R	4 (4.1)	2 (2.1)	0.683	2.04 (0.37–11.42)	7 (6.7)	10 (9.5)	0.448	0.68 (0.25–1.86)
C936T	21 (21.6)	29 (29.9)	0.189	0.65 (0.34–1.24)	37 (35.2)	42 (40.0)	0.476	0.82 (0.47–1.43)
A942S	7 (7.2)	10 (10.3)	0.446	0.68 (0.25–1.86)	8 (7.6)	12 (11.4)	0.347	0.64 (0.25–1.63)
A987G	11 (11.3)	2 (2.1)	0.010	6.08 (1.31–28.19)	5 (4.8)	1 (1.0)	0.210	5.20 (0.60–45.29)
T1055A	22 (22.7)	6 (6.2)	0.001	4.45 (1.72–11.54)	13 (12.4)	4 (3.8)	0.023	3.57 (1.12–11.33)
T1069A	0 (0.0)	0 (0.0)	/	/	5 (4.8)	0 (0.0)	0.070	/
T1071C	0 (0.0)	0 (0.0)	/	/	4 (3.8)	0 (0.0)	0.130	/

Abbreviations: CI, confidence interval; HCC, hepatocellular carcinoma; OR, odds ratio.

*Data are presented as *n* (%).

† Crude odds ratios were calculated using a two-by-two frequency table.

To further confirm the above results, a second independent set, including 105 HCC cases and 105 non-HCC controls from Qidong local hospitals, was applied. As shown in Table 3, the mutation rate of A799G (14.3% vs. 5.7%, *P = *0.038) and T1055A (12.4% vs. 3.8%, *P = *0.023) were again significantly higher in the HCC patients than the non-HCC controls, with respective crude ORs of 2.75 (95% CI: 1.02–7.39) and 3.57 (95% CI: 1.12–11.33); however, A987G failed to show a significant difference between the HCC and non-HCC patients (*P = *0.210). The linkage disequilibrium between A799G, A987G, and T1055A was analyzed by Haploview software (www.broad.mit.edu/mpg/haploview) according to the method described previously [25]. No evidence for linkage disequilibrium between any of these three mutations was found.

The three sets of data were then combined and subjected to a multivariate analysis under the unconditional logistic regression model. The combined data analysis indicated that the A799G, A987G, and T1055A mutations were independent factors significantly associated with HCC. After adjustments for HBeAg status, serum ALT level, and HBV-DNA level, A799G, A987G, and T1055A were associated with a 5.53-fold (95% CI: 1.69–18.10, *P = *0.005), 4.20-fold (95% CI: 1.15–15.35, *P = *0.030), and 3.78-fold (95% CI: 1.45–9.86, *P = *0.007) increased risk of HCC, respectively (Table 4). The positive predictive values of A799G, A987G, and T1055A were 0.732, 0.842, and 0.778, respectively. And the negative predictive values of A799G, A987G, and T1055A were 0.526, 0.517, and 0.535, respectively. In addition, compared to patients with an HBV-DNA level of <4 log_10_ copies/mL, the adjusted ORs of HCC for patients with HBV-DNA levels of 4 to <6 and ≥6 log_10_ copies/mL were 4.43 (95% CI: 2.03–9.69, *P*<0.001) and 1.14 (95% CI: 0.47–2.74, *P = *0.771), respectively (Table 4).

**Table 4:** Multivariate analysis of risk factors for hepatocellular carcinoma in all patients (n = 468).

Factor	Adjusted OR (95% CI)*	*P*
HBeAg		
Negative	1	
Positive	1.90 (0.91–3.99)	0.089
HBV DNA (log_10_ copies/mL)		
<4	1	
4–5.9	4.43 (2.03–9.69)	<0.001
≥6	1.14 (0.47–2.74)	0.771
ALT		
≤40 U/L	1	
>40 U/L	5.50 (2.83–10.68)	<0.001
A799G mutation		
Absence	1	
Presence	5.53 (1.69–18.10)	0.005
A987G mutation		
Absence	1	
Presence	4.20 (1.15–15.35)	0.030
T1055A mutation		
Absence	1	
Presence	3.78 (1.45–9.86)	0.007

Abbreviations: ALT, alanine aminotransferase; HBeAg, hepatitis B e antigen; HBV, hepatitis B virus; CI, confidence interval; OR, odds ratio.

*Adjusted odds ratios were estimated using an unconditional logistic regression analysis.

### Impact of T1055A and A799G Mutations on the HBV DNA Load

**Figure 3:**
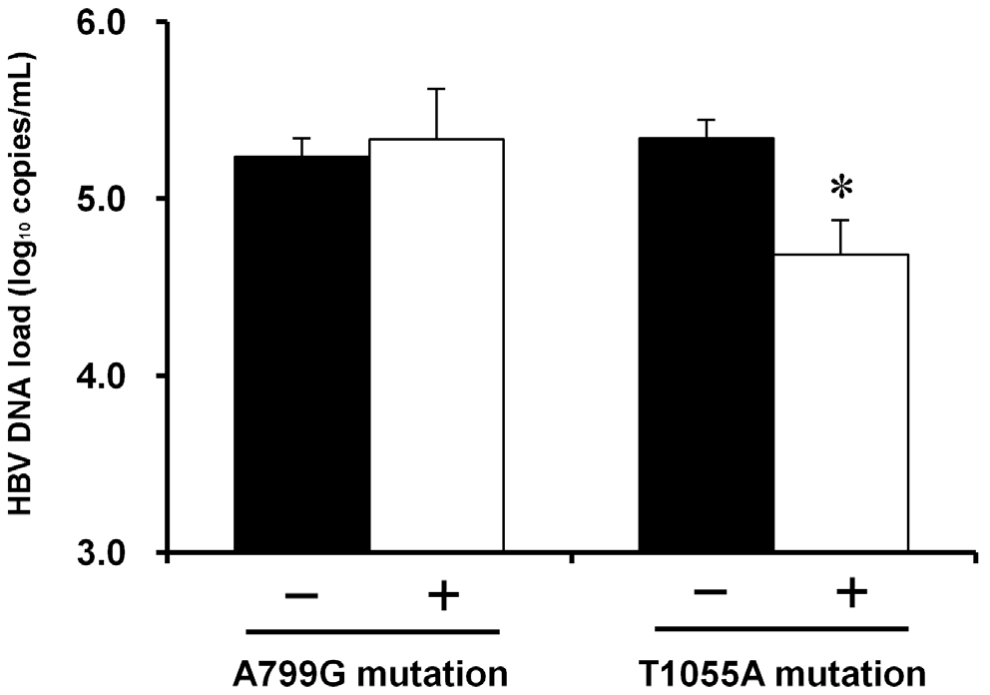
Effects of the A799G and T1055A mutations on circulating HBV DNA levels in all participants. The effects of the mutations A799G and T1055A on circulating HBV DNA load were estimated using blood samples from a total of 468 participants (HCC patients or non-HCC controls). Among these participants, 47 (10.0%) harbored A799G and 48 (10.3%) harbored T1055A. Circulating hepatitis B virus (HBV) DNA level was determined by quantitative real-time PCR. The data represent the mean ± SE. *, *P*<0.050.

The T1055A mutation causes a methionine to lysine substitution at rt309. Because the residues from rt304 to rt311 have been reported to be critical for RT activity [26], we analyzed the relationship between the rtM309K mutation and HBV DNA load. As shown in Figure 3, patients infected with the T1055A mutant had a significantly lower level of HBV DNA in their blood than those who were infected with prototype (4.69±1.19 vs. 5.34±1.58 log_10_ copies/mL, *P*<0.050). In contrast, the A799G mutation, a missense mutation that results in an isoleucine to valine substitution at the rt224 position did not affect the HBV DNA load in either the HCC or non-HCC patients (both *P*>0.050). These results suggested that impaired viral replication could be a consequence of the T1055A mutation, but not of the A799G mutation.

## Discussion

Numerous studies have shown that mutations in the HBV pre-S, BCP/EnhII, and pre-C regions are associated with the risk of HCC [4], [7], [27]–[34]. However, natural mutations in HBV RT and their relationship with HCC have rarely been explored before. In this study, we characterized spontaneous mutations in the HBV RT region and report, for the first time that the A799G, A987G, and T1055A mutations are independent risk factors for HCC. The association of these RT mutations with HCC is likely to be robust in genotype C-infected patients. Whether such mutations in other genotype of HBV have the similar association with HCC is an interesting issue for further study.

HBV RT performs the primary enzymatic function of viral replication and is the main target of anti-HBV drug development. Studies on RT mutations have consistently focused on the relationship with the resistance to NA treatment. The YMDD mutation in domain C is one of the common mutations in patients who are treated with lamivudine and entecavir. However, whether these mutants actually exist prior to antiviral therapy is debatable [35]–[38]. Our data indicated that the natural mutations at the common drug-related sites [12], [13], [24] were very rare, at least in Qidong where genotype C is prevalent. Among the 64 subjects in the discovery set whose full-length RT sequence were available, only one exhibited an entecavir-related mutation at rt169. None YMDD mutation was found in any of these cases. It is worth pointing out that the sequences used for RT comparison were isolated from the individuals residing in Qidong rural areas where NA treatment has not been introduced yet. Thus, our results presented a real natural mutation pattern of HBV RT. During the natural history of HBV infection, the spontaneous mutations were mainly distributed in the C-terminal region of RT, starting at the end of domain C. This is different from most of the drug-related mutations that are usually clustered inside domains B and C [13]. We speculate that naturally occurring mutations and NA-related mutations may belong to two distinct types of mutation and play different functional roles. The reason why some naturally occurring mutations in the C-terminal part of RT are selected during the development of HCC warrants further investigation.

The relationship between drug-resistance or RT domain mutations and HCC has yet to be elucidated. Our study revealed that the A799G and T1055A mutations have a significantly and consistently higher frequency in HCC patients than in non-HCC patients in both of the two validation sets. The multivariate analysis of the combined datasets showed that the presence of the A799G, A987G, and T1055A mutations was an independent risk factor for HCC development. Therefore, the association of these RT mutations with HCC is likely to be robust. A799G and T1055A are missense mutations that cause rtI224V and rtM309K aa substitutions, respectively, in RT. Based on three-dimensional modeling analysis, rt309 is located at the “turn” of the helix clamp motif in the thumb domain that interacts with the DNA template-primer. In this case, rtM309K aa substitution is likely to affect the precise conformation of the two α-helices and their interactions with the DNA template-primer, consequently impairing polymerase activity. The sequence from rt304 to rt311 is highly conserved among genotypes A–H, and cell transfection experiments indicated that natural or artificial substitutions in this region could drastically decrease the viral replicative competency [26], [39]–[41]. Further studies have revealed that the mechanisms for decreased replicative competency are mediated by hampering the encapsidation of pgRN [26], [41]. Interestingly, we also observed a significantly lower level of HBV DNA in the patients infected with rtM309K mutant in our study, providing further evidence from patients to support the important role of rt304–311 in viral replication. However the mechanism underling the association of rtM309K and HCC is not clear. *In vitro* and *in vivo* experiments using a 1.3× genome-length HBV construct containing rtM309K are needed to explore its biological impact on viral replication. Further, HBV transgenic mice containing RT mutated sequence could help to investigate the role of these mutants in liver carcinogenesis.

There are several studies from Asia reported that HBV viremia is associated with increased risk of HCC [34], [42]–[44]. However, few studies stratified the DNA level and reported a successively increased OR value of HCC with the viral DNA increasing [43], [44]. In the current study, the patients with middle-high level of serum HBV DNA (4–5.9 log_10_ copies/mL) were most likely to be associated with HCC. Our data are consistent with some recent publications [45]–[48]. Interestingly, these studies were all from east China, especially from Qidong area. So far, the explanation for this discrepancy is unclear. Different from other regions of Asia, almost all HBV patients (>90%) in Qidong are infected with genotype C [9], which was significantly associated with serum HBV DNA level [49]. Thus, HBV genotype may influence the association between HBV load and HCC risk.

Unlike the mutations A799G and T1055A, A987G does not cause an amino acid change in HBV polymerase. Interestingly, the nucleotide position 987 is close to the 5′ modulatory element of HBV enhancer I [50]. Enhancer I overlaps the promoter of the X gene, and has a dramatic effect on transcription of the HBx mRNA [51]. In spite of the lack of enhancer activity, the 5′ modulatory element can greatly increase the activity of the core domain of Enhancer I [52]. It is thus speculated that the mutation A987G might modulate the function of Enhancer I, and subsequently promote the transcription of HBx protein, which plays a role in the carcinogenesis of HCC [53]. Another possibility to explain the rationality of A987G is that this mutation may be in linkage disequilibrium with other well-defined HCC-related mutations such as pre-S deletion, the BCP/EnhII region mutations C1653T, T1753A/G, C1762T, and T1764A, and the precore mutations G1896A, G1899A. After the full-length HBV sequence information from all of the cases in this study is obtained, we will definitely perform the linkage disequilibrium analysis between A987G, as well as the other 2 identified RT mutations, and well-known HCC-related mutations.

As most HBV mutations occur during the course of chronic infection, it is important to determine the temporal relationship between HBV RT mutations and HCC occurrence. Therefore, we simultaneously examined the HBV RT sequence from samples collected 4–5 years prior to HCC diagnosis of the same patient. We found that the mutations A799G, A987G, and T1055A were detectable in >75% patients (3 of 4 patients for A799G, 2 of 2 patients for A987G, and 4 of 5 patients for T1055A) 4–5 years before the clinical diagnosis of HCC (Table S3 in File S1). No reverse mutations were observed at these sites. The persistence of such mutants in CHB patients suggests a potential causative role of RT mutation in the development of HCC. However, this requires further validation in large-scale cohort studies. As most of the current tumor markers, such as α-fetoprotein, only become elevated ∼approximately six months before the onset of HCC [54], our finding provided an important clue to HCC prediction, and suggested that the surveillance of these novel RT mutations, either independently or combined with other well-defined HCC-related mutations of HBV in pre-S, pre-C and BCP/Enh II, will benefit those who are at high risk of HCC with regard to early prevention and intervention.

## Supporting Information

File S1 Table S1, List of the primers used in the study. Table S2, Distribution of naturally occurring amino acid substitutions in the different regions of the hepatitis B virus reverse transcriptase domain. Table S3, Longitudinal observation of A799G, A987G, and T1055A mutations during hepatocellular carcinoma development.
(DOC)
