# Supplementary material for: Novel Natural Mutations in the Hepatitis B Virus Reverse Transcriptase Domain Associated with Hepatocellular Carcinoma
Source: PLoS One. 2014 May 1;9(5):e94864. doi: 10.1371/journal.pone.0094864 (PMC4006920; doi:10.1371/journal.pone.0094864)
Supplement: File S1 — Table S1, List of the primers used in the study. Table S2, Distribution of naturally occurring amino acid substitutions in the different regions of the hepatitis B virus reverse transcriptase domain. Table S3, Longitudinal observation of A799G, A987G, and T1055A mutations during hepatocellular carcinoma development. (DOC) [file pone.0094864.s001.doc]

**Supporting Information**

**Table S1. List of the primers used in the study**

| Primer name | Sequence | Nucleotide position |
| --- | --- | --- |
| HBV57F | 5'-CTGCTGGTGGCTCCAGTTC-3' | nt. 57-75 |
| FLP6 | 5'-TGGCTCAGTTTACTAGTGCCA-3' | nt. 668-688 |
| HBV696F | 5'-AGTGGTTCGTAGGGCTTTCC-3' | nt.696-715 |
| 1281R | 5'-GAGTTCCGCAGTATGGATCG-3' | nt.1281-1262 |
| 1193R2 | 5'-GCGTCAGCAAACACTTGGCA-3' | nt. 1193-1174 |

**Table S2.** Distribution of naturally occurring amino acid substitutions in the different regions of the hepatitis B virus reverse transcriptase domain

|  |  |  | HCC (*n* = 29) |  | Non-HCC (*n* = 35) |  | Total (*n* = 64) |  |
| --- | --- | --- | --- | --- | --- | --- | --- | --- |
| RT domain | Nucleotide position | Amino acid position | Substitution No. (%) | Frequency (per 1000 aa) | Substitution No. (%) | Frequency (per 1000 aa) | Substitution No. (%) | Frequency (per 1000 aa) |
| Space start-G | nt. 130-201 | rt. 1-24 | 1 (2.5) | 2.76 | 3 (5.8) | 3.43 | 4 (5.0) | 3.13 |
| Domain G | nt. 202-237 | rt. 25-36 | 0 (0.0) | 0 | 0 (0.0) | 0.00 | 0 (0.0) | 0 |
| Domain F | nt. 238-270 | rt. 37-47 | 1 (2.5) | 3.13 | 0 (0.0) | 0.00 | 1 (1.25) | 1.42 |
| Space F-A | nt. 271-351 | rt. 48-74 | 1 (2.5) | 2.55 | 3 (5.8) | 3.17 | 4 (5.0) | 2.89 |
| Domain A | nt. 352-402 | rt. 75-91 | 1 (2.5) | 2.03 | 3 (5.8) | 5.04 | 4 (5.0) | 3.68 |
| Space A-B | nt. 403-615 | rt. 92-162 | 8 (20.0) | 6.31 | 13 (25.0) | 6.44 | 20 (25) | 6.38 |
| Domain B | nt. 616-696 | rt. 163-189 | 2 (5.0) | 3.83 | 6 (11.5) | 6.35 | 7 (8.8) | 5.21 |
| Space B-C | nt. 697-726 | rt. 190-199 | 1 (2.5) | 3.45 | 0 (0.0) | 0.00 | 1 (1.25) | 1.56 |
| Domain C | nt. 727-759 | rt. 200-210 | 0 (0.0) | 0 | 1 (1.9) | 2.60 | 1 (1.25) | 1.42 |
| Space C-D | nt. 760-816 | rt. 211-229 | 3 (7.5) | 18.15 | 2 (3.8) | 3.01 | 5 (6.3) | 9.87 |
| Domain D | nt. 817-852 | rt. 230-241 | 3 (7.5) | 11.49 | 1 (1.9) | 4.76 | 3 (3.8) | 7.81 |
| Space D-E | nt. 853-867 | rt. 242-246 | 1 (2.5) | 13.79 | 1 (1.9) | 22.86 | 1 (1.25) | 18.75 |
| Domain E | nt. 868-900 | rt. 247-257 | 2 (5.0) | 40.75 | 1 (1.9) | 18.18 | 2 (2.5) | 20.41 |
| Space E-end | nt. 901-1161 | rt. 258-344 | 16 (40) | 14.27 | 18 (34.6) | 16.75 | 27 (33.8) | 18.86 |
| Full length | nt. 130-1161 | rt. 1-344 | 40 (100) | 9.01 | 52 (100) | 7.35 | 80 (100) | 7.81 |

Abbreviations: aa, amino acid; HCC, hepatocellular carcinoma; RT, reverse transcriptase

**Table S3.** Longitudinal observation of A799G, A987G, and T1055A mutations during hepatocellular carcinoma development

|  | 4-5 years before HCC onset |  |  |  | Diagnosis of HCC |  |  |
| --- | --- | --- | --- | --- | --- | --- | --- |
| Patient ID. | A799G | A987G | T1055A |  | A799G | A987G | T1055A |
| # 252 | ● | ○ | ○ |  | ● | ○ | ○ |
| # 3-073 | ● | ● | ○ |  | ● | ● | ○ |
| # 6-177 | ● | ○ | ○ |  | ● | ○ | ○ |
| # 99 | ○ | ○ | ○ |  | ● | ○ | ○ |
| # 195 | ○ | ● | ● |  | ○ | ● | ● |
| # 1-117 | ○ | ○ | ● |  | ○ | ○ | ● |
| #372 | ○ | ○ | ● |  | ○ | ○ | ● |
| # 6-008 | ○ | ○ | ● |  | ○ | ○ | ● |
| # 5-004 | ○ | ○ | ○ |  | ○ | ○ | ● |

Abbreviations: HCC, hepatocellular carcinoma.

Note: ○, wild type; ●, mutation.
